# Thyroid Cancer: Pathogenesis, Clinicopathology, Diagnosis, and Management

**DOI:** 10.1002/mco2.70449

**Published:** 2025-10-28

**Authors:** Yu‐Dong Li, Qing‐Yao Ye, Yan‐Xing Chen, Xia‐Rong Hu

**Affiliations:** ^1^ Department of Thyroid Surgery The Tenth Affiliated Hospital Southern Medical University (Dongguan People's Hospital) Dongguan Guangdong China

**Keywords:** thyroid cancer, differentiated thyroid cancer, medullary thyroid cancer, anaplastic thyroid cancer, thyroid‐stimulating hormone, ultrasound

## Abstract

Thyroid cancer is the most common endocrine malignancy, with incidence rising steadily worldwide. Although most cases are differentiated thyroid carcinomas with excellent prognosis, a small subset, such as anaplastic thyroid cancer, demonstrates aggressive behavior and poor survival outcomes. Recent decades have witnessed a transformation in thyroid cancer diagnostics and management, driven by improvements in high‐resolution ultrasound, fine‐needle aspiration biopsy, molecular profiling, and standardized risk stratification systems such as the Thyroid Imaging Reporting and Data System. Despite these advances, overdiagnosis and overtreatment remain key clinical challenges. Accurate risk stratification and management strategies are critical, especially for distinguishing indolent nodules from aggressive subtypes. This review provides a comprehensive overview of thyroid cancer pathogenesis, clinicopathological classification, diagnostic approaches, and evolving therapeutic strategies, ranging from active surveillance to targeted and immunotherapy‐based treatments. By integrating molecular diagnostics with conventional parameters, the article underscores how precision medicine can reduce treatment burden, improve outcomes, and guide personalized care. This review offers valuable insight into the biological complexity of thyroid cancer and highlights the need for continued refinement of diagnostic criteria and therapeutic frameworks in clinical practice.

## Graphical abstract

Thyroid cancer risk is influenced by both genetic and modifiable factors, including mutations, radiation exposure, lifestyle, and pre‐existing benign conditions. Diagnosis involves physical exams, ultrasound, scintigraphy, laboratory tests, and fine‐needle aspiration biopsy (FNAB). Management options range from active surveillance and surgery to advanced therapies like targeted treatments and immunotherapy. Future research focuses on refining targeted and combination therapies to improve outcomes. This multifaceted approach highlights the importance of early detection and personalized treatment strategies.

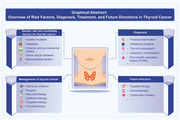

## 1 Introduction

Thyroid cancer has transitioned from being a rare malignancy to the most common cancer of the endocrine system. In recent decades, its incidence has risen steadily across the globe. According to the 2022 Global Cancer Statistics released by the International Agency for Research on Cancer, thyroid cancer ranked seventh among all malignancies worldwide, with more than 821,000 new cases, and was the fifth most frequently diagnosed cancer in women. The disease demonstrates a marked sex disparity, with a female‐to‐male ratio of approximately 3:1. Despite this high incidence, thyroid cancer generally has a favorable prognosis, contributing to around 44,000 deaths in 2022 and ranking only 24th among cancer‐related causes of mortality [1]. The cancer affects individuals of all ages, with a median age at diagnosis of 51 years. The highest incidence occurs between the ages of 45 and 64 years [2]. Of particular concern is its status as the most frequently diagnosed cancer among adolescents and young adults under the age of 40 years [3, 4]. In China, thyroid cancer has emerged as the most rapidly increasing malignancy. Between 2003 and 2011, the annual growth rate of incidence was approximately 20%, the fastest among all cancer types in the country [5]. Age‐standardized incidence rates were estimated at 16.8 per 100,000 women and 5.3 per 100,000 men, demonstrating a similar gender disparity to global patterns [6]. However, substantial regional variation exists within China, with some provinces reporting incidence rates up to 45 times higher than others [7]. In contrast to its rapidly growing incidence, thyroid cancer mortality in China has remained low and stable, a trend that mirrors international observations. [7, 8] For example, in 2010, the age‐standardized mortality rate was just 0.35 per 100,000 women and 0.19 per 100,000 men [6, 7]. These figures produce exceptionally high incidence‐to‐mortality ratios, exceeding 40 years in women and 20 years in men, which reinforces the generally indolent nature of most thyroid cancers.

Although most thyroid cancers, particularly differentiated thyroid carcinomas (DTCs), are biologically indolent and associated with excellent long‐term survival, the clinical management of thyroid nodules presents complex challenges. The widespread adoption of high‐resolution ultrasonography has led to an increase in the detection of small, often subclinical, thyroid nodules [9, 10]. Current estimates suggest that approximately 90% of identified nodules are benign, and more than half of thyroid cancers remain small, localized, and confined to the thyroid gland at the time of diagnosis [11, 12, 13, 14]. This phenomenon has resulted in what is now termed an “epidemiologic paradox,” wherein the incidence of thyroid cancer continues to rise while mortality remains unchanged. This increase in detection, however, comes at a cost [11, 15]. A growing body of evidence points to significant overdiagnosis and overtreatment of thyroid nodules that may never progress to cause clinical harm. From a health economics perspective, the management of thyroid cancer generates a disproportionately high financial burden [16]. Patients often undergo repeated imaging, fine‐needle aspiration biopsies (FNAB), total thyroidectomy, radioactive iodine (RAI) ablation, and lifelong thyroid hormone replacement therapy [11, 15]. These interventions, although effective, may be unnecessary for low‐risk patients and have been linked to higher rates of bankruptcy and financial toxicity among survivors compared with patients with other types of cancer [11, 15]. To address this challenge, accurate risk stratification is essential. Diagnostic strategies now incorporate structured systems such as the Thyroid Imaging Reporting and Data System (TI‐RADS), which uses ultrasound characteristics to classify nodules by their likelihood of malignancy [12, 17, 18, 19, 20]. High‐suspicion nodules are typically further evaluated using FNAB, which provides cytologic classification and guides clinical management [12, 21]. In parallel, molecular diagnostics have advanced significantly in recent years, offering improved diagnostic accuracy for indeterminate nodules. These tools enhance risk stratification by identifying genetic mutations and molecular signatures associated with tumor aggressiveness. Traditional management of thyroid cancer was centered on a uniform approach that included surgical resection and postoperative RAI therapy. This model served well for many years, particularly for high‐risk or metastatic disease. However, accumulating data from observational studies and randomized trials have prompted a reassessment of this strategy. In particular, low‐risk papillary thyroid microcarcinomas are now recognized as clinically indolent in many cases. As a result, active surveillance (AS) has gained acceptance as a viable initial management option for select patients with small, low‐risk tumors. This strategy involves regular follow‐up and imaging without immediate intervention and has been shown to reduce overtreatment without compromising oncologic outcomes [22, 23]. In addition to surveillance, nonsurgical, minimally invasive treatment options such as radiofrequency ablation (RFA) and high‐intensity focused ultrasound (HIFU) have gained momentum in clinical practice [24, 25]. These techniques offer effective symptom relief and local tumor control, with fewer complications and shorter recovery times compared with conventional surgery. Minimally invasive therapies are particularly attractive for patients with comorbidities or contraindications to surgery and for those who prefer organ‐preserving approaches. The most significant recent advance in thyroid cancer treatment has been the emergence of precision medicine. Molecular diagnostics now enable the identification of actionable mutations such as BRAF V600E, TERT promoter mutations, RET rearrangements, and NTRK fusions [26]. These findings have led to the development and clinical use of targeted therapies, including tyrosine kinase inhibitors and immune checkpoint inhibitors, which offer improved outcomes for patients with advanced, progressive, or treatment‐refractory disease [26]. These agents have demonstrated efficacy in cases previously considered untreatable and underscore the importance of integrating genetic profiling into routine clinical decision‐making. Despite significant advancements in the understanding, diagnosis, and treatment of thyroid cancer, numerous challenges persist in clinical practice. The increasing burden of incidental diagnoses, financial strain on patients, variability in diagnostic interpretation, and disparities in access to molecular testing all contribute to the complexity of thyroid cancer care. Clinicians must strike a careful balance between minimizing overtreatment and ensuring timely intervention for aggressive disease.

This review aims to provide a comprehensive and updated synthesis of thyroid cancer research, focusing on key aspects such as epidemiology, risk factors, diagnostic approaches, histological and molecular classification, treatment modalities, and emerging therapies. By integrating conventional diagnostic methods with next‐generation molecular profiling and precision treatment strategies, this article highlights the evolving landscape of thyroid cancer management. The goal is to offer a clinically relevant overview that supports evidence‐based, patient‐centered care and identifies opportunities for future research and clinical innovation.

## 2 Etiology of Thyroid Cancer

### 2.1 Genetic Risk Factors

**FIGURE 1:**
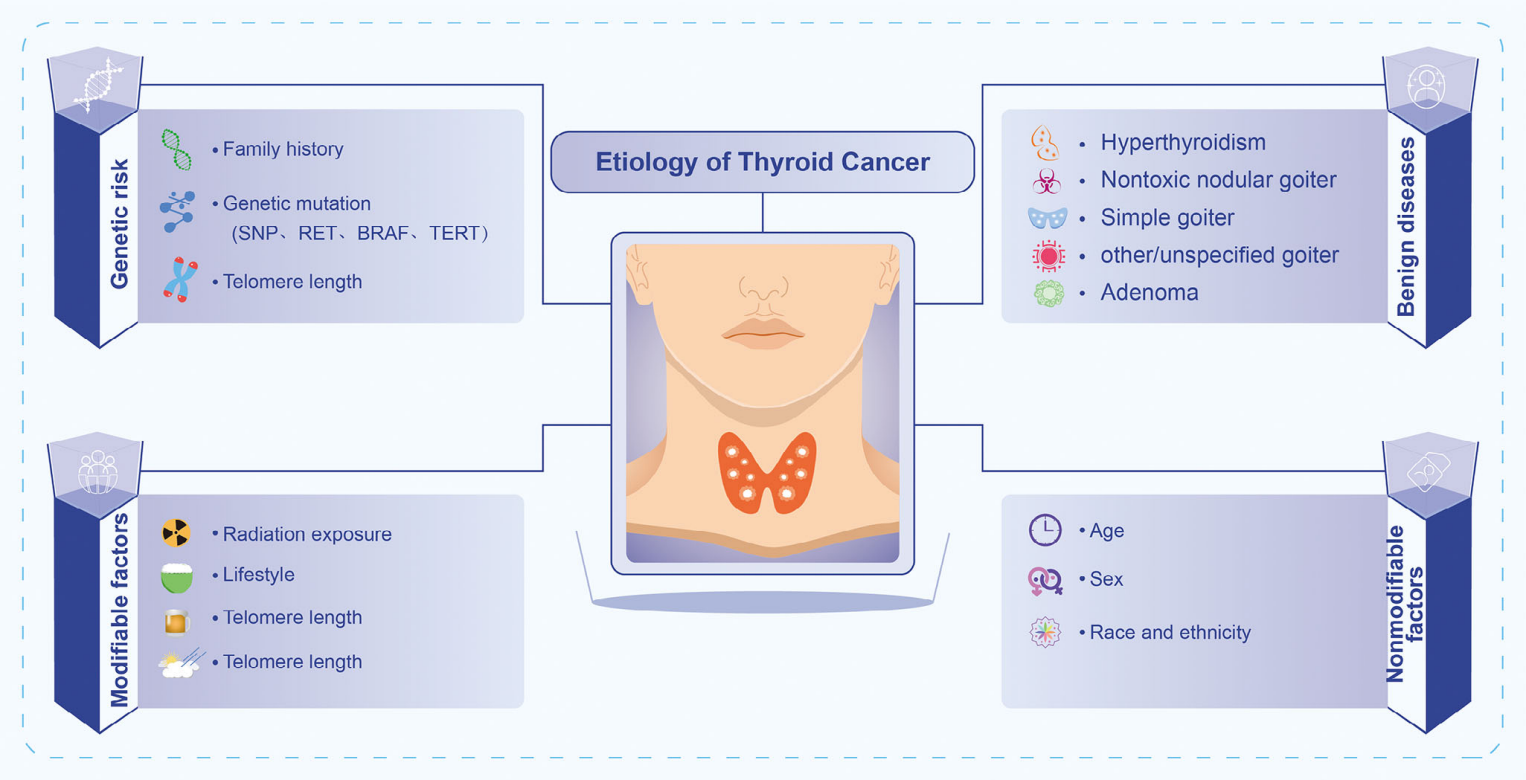
Etiology of thyroid cancer. Thyroid cancer risk involves genetic factors, nonmodifiable traits, and environmental exposures, lifestyle, and benign thyroid conditions. Recognizing these factors aids prevention and early detection.

Thyroid carcinogenesis results from complex interactions between genetic predisposition and environmental exposures (Figure 1). Among the 15 most common cancers, thyroid cancer demonstrates the highest estimated heritability, at approximately 53% [27]. Medullary thyroid carcinoma (MTC) arising from parafollicular C cells exhibits hereditary patterns in 20–25% of cases [28, 29]. In contrast, non‐MTCs, particularly DTCs such as papillary thyroid carcinoma (PTC) and follicular thyroid carcinoma (FTC), also demonstrate familial clustering, with up to 9% of cases having a first‐degree relative affected [30]. Genome‐wide association studies have uncovered novel susceptibility loci for thyroid cancer. A multicenter study involving 3001 non‐MTC patients and 287,550 controls identified five new risk loci through whole‐genome sequencing [31]. Moreover, several single‐nucleotide polymorphisms have been associated with increased PTC risk [32]. Emerging data also highlight telomere length as a potential biomarker for thyroid cancer susceptibility [33].

### 2.2 Modifiable Risk Factors

Ionizing radiation remains one of the most well‐established environmental risk factors for thyroid cancer [34]. The first documented link between childhood radiation exposure and thyroid malignancy was reported in 1950, when 10 out of 28 pediatric patients with thyroid cancer had undergone radiotherapy [35]. Data from post‐Chernobyl surveillance further support a dose‐dependent relationship: children exposed to RAI demonstrated a significantly elevated incidence of thyroid cancer, with a sharp increase in risk beyond 40 Gy [34, 36, 37, 38]

Lifestyle and environmental factors also play a significant role in thyroid cancer risk. A large prospective study showed that individuals with unfavorable lifestyle patterns, including poor diet, physical inactivity, obesity, smoking, and alcohol consumption, had nearly double the risk of developing thyroid cancer (HR, 1.93; 95% CI, 1.50–2.49; *p* < 0.001) [39]. Obesity, in particular, is strongly correlated with thyroid cancer incidence. Meta‐analyses reveal a dose–response relationship, with each 5 kg/m^2^ increase in body mass index (BMI) associated with a 6–30% increased risk of thyroid malignancy [40]. Other potential environmental contributors include chronic stress, night shift work, proximity to volcanic regions, and pesticide exposure [41].

### 2.3 Association with Benign Thyroid Disorders

Several benign thyroid conditions are associated with an increased risk of malignant transformation. A nationwide cohort study reported that patients with benign thyroid diseases, including hyperthyroidism, nontoxic nodular goiter, simple goiter, unspecified goiter, and thyroid adenoma, exhibit substantially higher risks of thyroid cancer [42]. Furthermore, elevated thyroid‐stimulating hormone (TSH) levels, even within the normal reference range, have been implicated in increasing DTC risk [43, 44, 45, 46]. However, this correlation appears to be absent in patients with autoimmune thyroid disease, suggesting that TSH may exert distinct effects in different pathological contexts [47].

### 2.4 Nonmodifiable Factors

Nonmodifiable demographic factors such as age, sex, race, and ethnicity also influence thyroid cancer incidence and outcomes [4]. According to the SEER‐9 (Surveillance, Epidemiology, and End Results) registry data from 1974 to 2013, women exhibit a threefold higher incidence of thyroid cancer compared with men [48]. Advanced age is associated with both increased incidence and worse prognosis. For example, individuals aged 60–79 years exhibit an incidence rate of 13.15 per 100,000 and a mortality rate of 0.22 per 100,000, substantially higher than the rates seen in younger individuals [48].

In summary, thyroid cancer arises from a multifactorial interplay of genetic susceptibility, environmental exposures (especially ionizing radiation), lifestyle influences, hormonal factors, and pre‐existing benign thyroid conditions. Understanding these risk determinants is essential for guiding targeted prevention strategies and personalized screening protocols.

## 3 Diagnosis of Thyroid Cancer

An ideal diagnostic strategy for thyroid cancer aims to maximize cost effectiveness, reduce unnecessary invasive procedures, enhance accuracy in identifying clinically significant malignancies, and streamline personalized treatment planning. Over the past few decades, substantial progress in pathological classification, imaging modalities, molecular techniques, and biomarker development has dramatically improved diagnostic performance. Traditionally, thyroid cancer was identified by the detection of a palpable thyroid nodule [49, 50, 51]. Today, diagnosis is typically based on a combination of physical examination, imaging, laboratory tests, cytopathology, and molecular diagnostics.

### 3.1 Physical Examination

Thyroid nodules are often identified incidentally during routine physical examination or imaging performed for unrelated conditions. Palpation alone detects approximately 4–7% of nodules [52]. However, imaging modalities reveal a far greater prevalence: ultrasound identifies 20–67% of nodules, contrast‐enhanced computed tomography (CT) up to 25%, magnetic resonance imaging (MRI) 10–18%, and positron emission tomography (PET) 2–2.3% [53, 54, 55]. In some cases, nodules cause compressive symptoms such as hoarseness, dysphagia, dyspnea, or hemoptysis, often due to local invasion. Cervical lymphadenopathy, particularly in the lateral neck, may indicate metastatic disease. A thorough evaluation of these symptoms and clinical signs is crucial for early detection and staging.

### 3.2 Ultrasound Imaging

**FIGURE 2:**
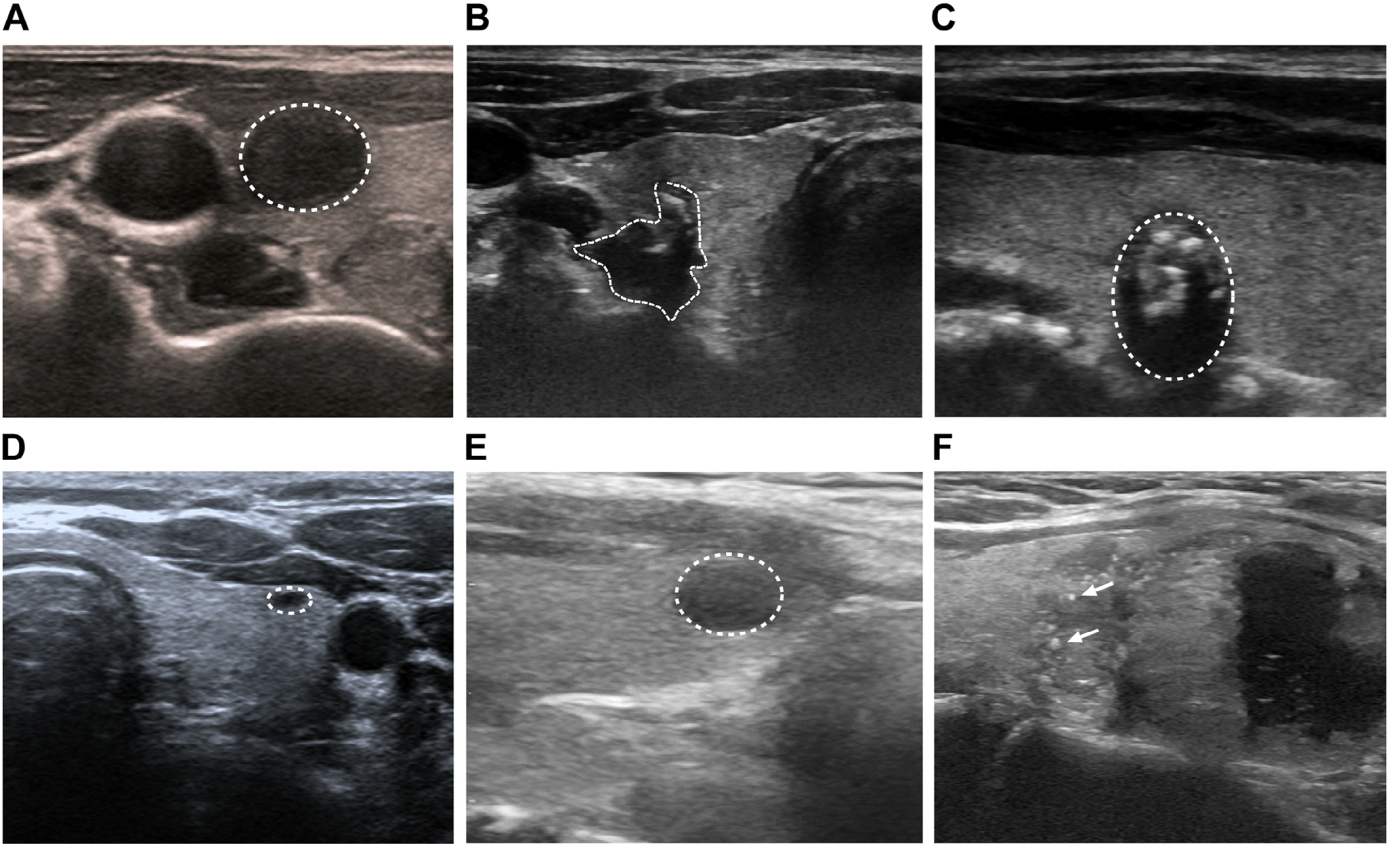
The features of ultrasound imaging. (A) Regular margin; (B) irregular margin; (C) taller‐than‐wide shape: the anteroposterior diameter is greater than its transverse diameter; (E) hypoechogenicity; (F) medium echogenicity; (G) punctate hyperechoic foci.

Ultrasound imaging is the cornerstone of thyroid nodule evaluation due to its noninvasive nature, accessibility, and high sensitivity. It provides detailed information on nodule echogenicity, shape, margins, internal composition, and the presence of calcifications (Figure 2 and Table S1) [56]. High‐resolution cervical ultrasound is recommended as the first‐line imaging modality for any detected or suspected thyroid lesion. However, no single ultrasound feature is pathognomonic for malignancy. Diagnostic accuracy heavily depends on the sonographer's expertise, and interobserver variability remains a significant limitation [57]. To address this, several professional societies have developed standardized risk stratification systems (RSSs) incorporating distinct sonographic features to guide the need for FNAB. Widely used systems include: American College of Radiology Thyroid Imaging Reporting and Data System (ACR‐TIRADS) [58], TIRADS developed by Kwak et al. (Kwak‐TIRADS) [59], the 2020 Chinese Guidelines for Ultrasound Malignancy Risk Stratification of Thyroid Nodules (C‐TIRADS) [60], the European Thyroid Association (EU‐TIRADS) [61], the American Thyroid Association (ATA) guidelines [62], the American Association of Clinical Endocrinologists, American college of Endocrinology, and Association Medici Endocrinology (AACE/ACE/AME) [63], and the Korean Thyroid Imaging Reporting and Data System (K‐TIRADS) [64]. Meta‐analyses suggest that ACR‐TIRADS provides the highest diagnostic accuracy among current systems [65]. Additionally, Kwak‐TIRADS and C‐TIRADS show strong performance in distinguishing indeterminate cytology, such as atypia of undetermined significance or follicular lesion of undetermined significance nodules [66]. Despite their utility, RSSs lack standardization in terminology and often omit nonsonographic clinical factors, such as age or family history. To address these gaps, the Italian Thyroid Cancer Observatory proposed a novel scoring system that integrates demographic and clinical variables such as age, BMI, tumor size, sex, family history of thyroid cancer, surgical approach, presurgical cytology, and circumstances of the diagnosis [67]. Additionally, the International Thyroid Nodule Ultrasound Working Group has initiated the development of a unified global lexicon and malignancy risk assessment system known as International Thyroid Imaging Reporting and Data System (I‐TIRAD) [68]. Future improvements should focus on reducing interobserver variability, incorporating nonimaging clinical variables into risk models, and validating suspicious features such as the “taller‐than‐wide” shape, which recent data suggest may not independently predict malignancy [69].

### 3.3 Thyroid Scintigraphy

**FIGURE 3:**
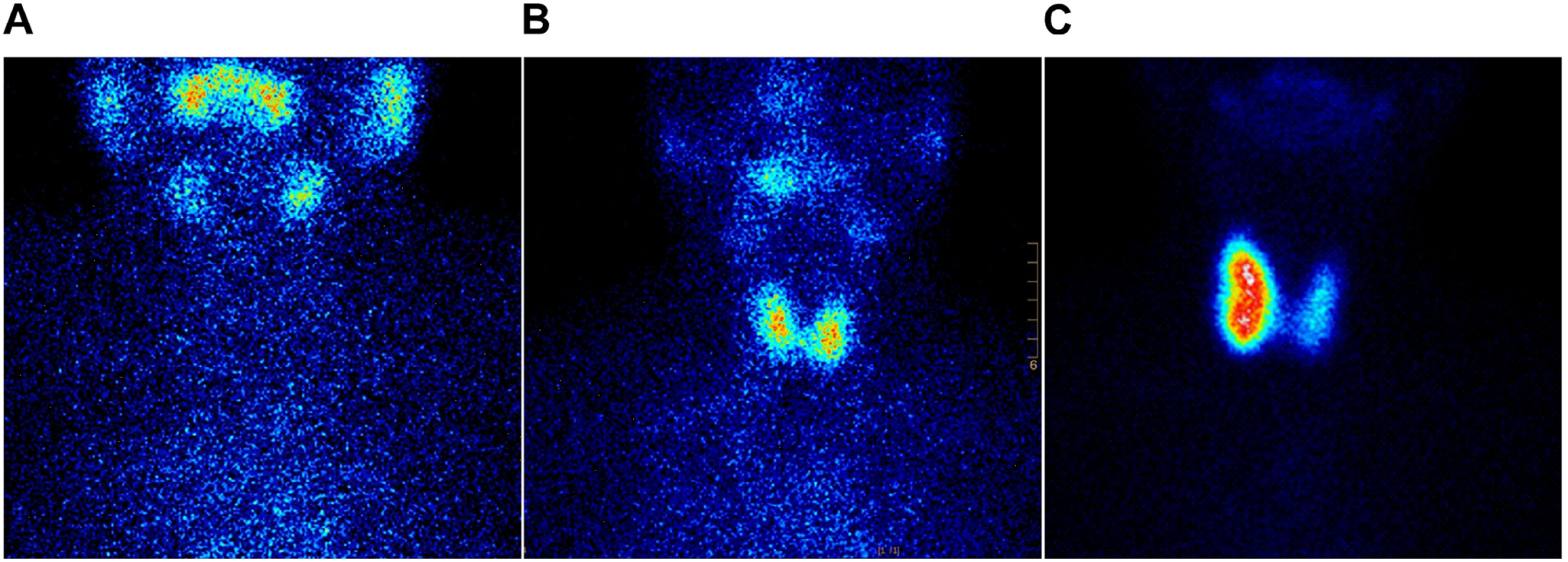
The figures of thyroid scintigraphy. We used ^99m^TcO_4_Na as radiotracer. The imaging equipment specifications including physical half‐life: 6.02 h, imaging time: 20 min, *Y*‐ray energy (keV): 140, and dose (MBq): 74–185. The figures of thyroid scintigraphy. (A) Cold nodule: the radiological distribution of nodules is sparse and defective; (B) warm nodules: the radioactive distribution of nodules is moderately average; (C) hot nodule: the radioactive distribution of nodules is concentrated.

While cross‐sectional imaging modalities such as ultrasound, CT, and MRI provide excellent anatomical detail of the thyroid gland [70, 71], thyroid scintigraphy uniquely evaluates nodule functionality. It quantifies metabolic activity based on ^123^I or _99m_ Tc uptake kinetics, offering functional insights not achievable with anatomic imaging alone. Although high‐resolution ultrasound has largely replaced thyroid scintigraphy in routine practice, the latter remains clinically valuable in specific scenarios, especially for evaluating nodules with suppressed TSH levels or in multinodular goiters. Scintigraphy classifies nodules into hyperfunctioning (“hot”), iso‐functioning (“warm”), and hypo‐functioning (“cold”) based on iodine uptake (Figure 3 and Table S1). “Cold” nodules are associated with a higher risk of malignancy and often warrant FNAB, whereas “hot” nodules are typically benign but may require additional evaluation if symptomatic or large [72]. Importantly, factors such as amiodarone therapy, antithyroid medications, or recent iodinated contrast exposure can suppress thyroid uptake and confound scintigraphy interpretations.

### 3.4 Laboratory Investigations

The primary role of laboratory investigations in thyroid cancer is to evaluate thyroid function, identify biochemical markers of malignancy, and monitor for recurrence. The most frequently employed serum markers include TSH, thyroglobulin (Tg), anti‐Tg antibodies (TgAb), calcitonin (Ctn), and carcinoembryonic antigen (CEA).

#### 3.4.1 TSH Measurement

TSH measurement is the initial test of choice when evaluating a thyroid nodule. In the presence of subnormal TSH levels, further investigation is warranted, including measurement of free thyroxine and free triiodothyronine levels, and a radionuclide thyroid scan to distinguish between autonomous (hot) and nonfunctioning (cold) nodules [10, 62].

While low TSH typically reflects autonomous thyroid hormone production, the threshold for functional autonomy varies based on regional iodine status. In iodine‐deficient populations, autonomously functioning nodules may be observed even within the low‐normal TSH range [73, 74].

#### 3.4.2 Tumor Markers

Tg, a thyroid‐specific glycoprotein, is a reliable postoperative surveillance marker but lacks specificity for preoperative diagnosis. Elevated Tg levels after total thyroidectomy or lobectomy may signal disease recurrence or residual tissue [75, 76]. However, the presence of TgAb in up to 25% of DTC patients interferes with Tg quantification, necessitating simultaneous TgAb testing [77, 78]. In rare cases where tumor cells fail to synthesize Tg, the marker becomes ineffective. Recent studies have also explored CD8+ T‐cell immune‐profiling as a prognostic biomarker for PTC recurrence, with effector memory subsets showing high predictive value [79].

Ctn is indispensable for the diagnosis and follow‐up of MTC. In most cases, serum Ctn levels correlate with tumor burden [80, 81]. Nevertheless, elevated Ctn can occur in non‐MTC conditions, such as chronic renal failure, sepsis, and other neuroendocrine tumors, complicating interpretation [82]. Universal diagnostic cutoffs remain undefined, limiting routine screening in nodular thyroid disease. CEA is elevated in 60–70% of MTCs and is useful as a prognostic biomarker, although it lacks specificity due to expression in other malignancies and benign conditions [83, 84]. Additional markers like carbohydrate antigen 19‐9 and procalcitonin have shown promise in small studies [85, 86, 87].

Emerging biomarkers such as circulating tumor DNA (ctDNA) and microRNAs (miRNAs) are being investigated for their diagnostic and surveillance potential in both DTC and MTC [88, 89]. NGS platforms enable the identification of actionable mutations (e.g., *BRAF V600E*, *TERT* promoter, *RET*), which guide targeted therapy and enhance diagnostic precision [90].

Despite progress, variability in assay techniques, sensitivity, and interpretation remains a challenge. Standardization efforts are crucial to ensure the reproducibility and clinical utility of serum and molecular markers.

### 3.5 Fine‐Needle Aspiration Biopsy

FNAB is the cornerstone for differentiating benign from malignant thyroid nodules. Ultrasound guidance enhances its diagnostic yield, particularly in nodules ≥1 cm exhibiting high‐risk features such as solid hypoechoic texture, microcalcifications, irregular margins, taller‐than‐wide shape, and extrathyroidal extension [58, 62]. However, FNAB is not universally indicated for all nodules, and some institutions adopt more conservative size thresholds. Cytologic results are reported using standardized systems, most notably the Bethesda System for Reporting Thyroid Cytopathology [91, 92], which is widely adopted in the United States. Other similar reporting systems have been employed in other areas; for example, a five‐tiered classification was proposed by the Italian Society for Anatomic Pathology and Cytology in collaboration with the Italian Division of the International Academy of Pathology [93]. Additionally, the 2019 Japanese reporting system for Thyroid Aspiration Cytology (JRSTAC2019) was developed by the Japan Association of Endocrine Surgery and the Japanese Society of Thyroid Pathology [94].

Cytological specimens demonstrating colloid and monolayered follicular cells with uniformly dispersed “salt‐and‐pepper” chromatin are diagnostically categorized as benign. Malignant features include nuclear pseudoinclusions, syncytial arrangements, and grooved nuclei [13]. Indeterminate cytology (Bethesda categories III and IV) poses a diagnostic dilemma. While repeat FNAB may be warranted, modern practice increasingly incorporates molecular testing to detect high‐risk mutations (e.g., *BRAF V600E*, *TERT* promoter), which predict recurrence and metastasis risk [95]. Conversely, nodules categorized as benign (Bethesda II) or malignant (Bethesda V/VI) generally do not require molecular testing for diagnostic purposes.

Looking forward, FNAB will continue to serve as a critical diagnostic modality, especially when integrated into multimodal diagnostic frameworks that include molecular profiling and advanced imaging techniques. This integrative strategy holds promise for enhancing diagnostic accuracy and minimizing overtreatment.

## 4 Histologic Subtypes and Molecular Profiling

**FIGURE 4:**
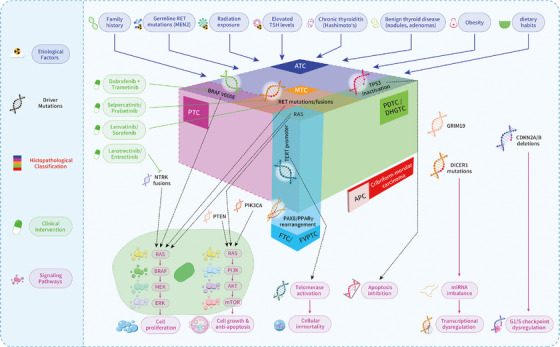
Molecular landscape of thyroid cancer pathogenesis. This figure presents a comprehensive overview of the key biological factors, driver mutations, and associated clinical implications in the development and progression of thyroid cancer, illustrating the complex interplay between genetic alterations, risk factors, signaling pathways, and cellular mechanisms that define different tumor subtypes and guide modern therapeutic strategies.

The fifth edition of the World Health Organization (WHO) Classification of Endocrine Tumors, released in 2022, represents a landmark revision in the categorization of thyroid neoplasms. This update reflects a deeper understanding of tumor biology by integrating morphological, immunohistochemical, and molecular data into a refined classification framework (Figure 4) [96]. The revised taxonomy stratifies thyroid tumors based on their cellular origin, growth behavior, and genetic alterations, enabling improved diagnostic precision, risk stratification, and therapeutic guidance.

A major advance in the current edition is the molecular subclassification of follicular‐derived tumors. Key molecular alterations, including *BRAF V600E*, *RAS* family mutations, *RET/PTC* rearrangements, and *PAX8/PPARγ* fusions, are now incorporated into subtype definitions. This approach enhances differentiation between tumor types and identifies candidates for targeted therapies, particularly in aggressive subtypes such as poorly differentiated thyroid carcinoma (PDTC) and anaplastic thyroid cancer (ATC).

In addition to molecular insights, the WHO 2022 classification redefines and expands specific tumor entities. For example, the category of “hyalinizing trabecular tumor” (HTT) is now recognized as a distinct entity based on its unique *RET::CCDC6* rearrangement. Similarly, “follicular thyroid adenoma with papillary architecture” is classified as a benign, distinct from PTC, due to its lack of nuclear features and differing molecular signature.

The updated guidelines also emphasize the use of immunohistochemical thyroid markers such as transcription factor 1 (TTF‐1), Tg, and BRAF V600E immunostaining, particularly in challenging diagnostic settings. Additionally, molecular markers like *TERT* promoter mutations and *TP53* mutations are recognized as key predictors of aggressive behavior and poor prognosis.

### 4.1 Follicular‐Derived Thyroid Tumors

#### 4.1.1 Benign Tumors

The fifth edition of the WHO classification of endocrine tumors categorizes follicular‐derived thyroid neoplasms into three groups: benign tumors, low‐risk neoplasms, and malignant neoplasms. Compared with previous editions, the 2022 update significantly broadens the spectrum of benign entities by incorporating molecular and architectural characteristics, addressing prior classification limitations. Historically, follicular adenoma was considered the prototypical benign entity, characterized as a well‐circumscribed, encapsulated tumor with typical follicular architecture and absent features of malignancy [97]. However, the term “multinodular goiter” remains pathologically ambiguous, as it encompasses a heterogeneous mixture of non‐neoplastic, hyperplastic, and neoplastic nodules. To address this, the WHO now recommends the term “thyroid follicular nodular disease” to describe these multifocal proliferative conditions [97].

Two additional benign entities are formally recognized: follicular adenoma with papillary architecture and the oncocytic adenoma. Follicular adenomas are encapsulated, noninvasive tumors with centripetal papillary structures confined within follicles. They lack nuclear features of PTC and often demonstrate autonomous function. Up to 70% harbor activating TSH receptor (*TSHR*) mutations, while smaller subsets show *GNAS* or *EZH1* mutations [96, 98, 99, 100, 101, 102, 103]. These mutations activate the cAMP pathway, promoting nodule growth independent of TSH signaling [104]. Oncocytic adenoma, formerly known as Hürthle cell adenoma, is composed of ≥75% oncocytic (oxyphilic) cells and shows no capsular or vascular invasion. Oncocytic adenomas often exhibit mitochondrial DNA mutations that impair oxidative phosphorylation, and a significant proportion show copy number alterations or mutations in mitochondrial complex I‐related gene (e.g., *GRIM19*/*NDUFA13*) [105, 106, 107, 108, 109, 110]. The term “Hürthle cell” is discouraged, as it historically referred to parafollicular C cells, not follicular‐derived oxyphilic cells.

#### 4.1.2 Low‐Risk Neoplasms

Low‐risk neoplasms occupy an intermediate position between benign and malignant tumors. The fifth WHO Classification of Endocrine Tumors (2022) delineates three entities within the spectrum of low‐risk follicular cell‐derived thyroid neoplasms: noninvasive follicular thyroid neoplasm with papillary‐like nuclear features (NIFTP), thyroid tumors of uncertain malignant potential (UMP), and HTT. These tumors exhibit minimal or no invasive behavior and generally lack metastatic potential, thus not requiring aggressive treatment [96].

NIFTP is defined as a well‐demarcated, encapsulated follicular‐patterned tumor with nuclear features resembling PTC, but without capsular/vascular invasion, tumor necrosis, or high mitotic activity [111, 112, 113, 114]. Complete histologic examination of the tumor capsule is essential to exclude invasive foci. Diagnostic criteria allow for ≤1% true papillae, provided *BRAF V600E* mutation is absent. NIFTPs are typically *RAS*‐like, harboring *RAS* mutations, *BRAF K601E*, or *THADA* and *PAX8::PPARG* fusions, but not *BRAF V600E* [115, 116, 117].

UMP refers to encapsulated or well‐demarcated tumors with uncertain capsular or vascular invasion after exhaustive sampling [97]. Based on nuclear scoring, UMPs are subclassified into follicular tumor of UMP (FT‐UMP) and well‐differentiated tumor of UMP (WDT‐UMP) [96, 118, 119, 120, 121, 122]. These tumors often share the same *RAS*‐like mutation spectrum as NIFTP.

HTT, on the other hand, is a rare lesion composed of trabecular nests of tumor cells with hyaline stroma and PTC‐like nuclear features. HTTs are uniquely characterized by *PAX8::GLIS1* or *PAX8::GLIS3* gene fusions and are now considered a distinct molecular and histologic entity [123, 124, 125, 126].

#### 4.1.3 Malignant Neoplasms

##### 4.1.3.1 FTC and Follicular Variant of PTC

FTCs, predominantly driven by *RAS* mutations, are characterized by invasive growth patterns without the nuclear features of PTC. According to the histological characteristics of capsule and vascular invasion, FTC is subclassified into three histopathological categories: minimally invasive FTC, encapsulated angio‐invasive FTC, and widely invasive FTC (Table 1). These classifications carry significant prognostic implications. In a prospective multicenter cohort (*n* = 124 FTC patients; median follow‐up 40 months), disease‐free survival rates varied significantly across histological subtypes: 97% for minimally invasive FTC, 81% for encapsulated angio‐invasive FTC, and 46% for widely invasive FTC, respectively [127]. The diagnostic paradigm for FVPTC has evolved substantially in recent years. The noninvasive encapsulated type has been reclassified as noninvasive follicular thyroid NIFTP. In the fifth edition of classification, follicular variant PTCs (FVPTCs) are further subdivided into “encapsulated with invasion” (Table 1) and “infiltrative” subtypes. The invasive encapsulated FVPTC is not incorporated into the PTC category and is considered an independent entity in malignant neoplasms, while the infiltrative form remains a PTC subtype. Histologically, the infiltrative subtype demonstrates classical PTC‐type invasion patterns but exhibits exclusively follicular architecture. Not only the histological characteristics, but also the molecular features guide the classification. *RAS*‐like mutations are characteristic of expansile growth patterns and subtle or less florid nuclear atypia, while *BRAF*‐like mutations are associated with infiltrative growth and florid nuclear atypia. Both noninvasive and invasive encapsulated FVPTCs typically exhibit *RAS*‐like mutations, whereas infiltrative follicular PTC subtypes show *BRAF*‐like mutations [128]. Besides, PTC also shows *BRAF*‐like mutations. Prognostically, invasive encapsulated FVPTC demonstrates significantly better outcomes compared with infiltrative subtypes [129]. These diagnostic refinements necessitate rigorous histopathological evaluation. Current guidelines mandate meticulous examination of tumor capsules and systematic documentation of invasion patterns. Of particular relevance, patients with widely invasive encapsulated FVPTC demonstrate substantial metastatic potential, and aggressive therapy might be suitable for these patients [130].

**TABLE 1:** Summary of malignant thyroid neoplasms from follicular cell (WHO fifth edition).

Tumor type	Histopathological categories	Molecular drivers	Histopathological features	Prognosis and clinical behavior
FTC	Minimally invasion Encapsulated angio‐invasion Wide invasion	*RAS* [96] *PAX8::PPARG* rearrangements [131] Other mutation: *PTEN*, *PIK3CA*, *DICER1*, *DGCR8*, *TERT* promoter [132, 133, 134, 135, 136, 137]	Follicular architecture No PTC nuclei	DFS: 97% (minimally invasion) 81% (encapsulated angio‐invasion) 46% (widely invasion) [127] Distant metastasis: Primarily occur in lung/bone, also in liver, brain, and other sites [138, 139, 140]
IEFVPTC	Similar to FTC	Similar to FTC	Follicular growth pattern PTC nuclei	Similar to FTC [141]
PTC	Classical, infiltrative follicular, tall cell, columnar cell, hobnail, solid, diffuse sclerosing, warthin‐like, and oncocytic PTC [96]	*BRAF* [128] *TERT* promoter [134, 142, 143, 144, 145, 146, 147, 148] *RET*/*NTRK1‐3*/*BRAF*/*ALK* fusion [149, 150, 151, 152, 153, 154] *PLEKHS1*, *TP53*, *RAS* [155]	Papillary structures, presenting clear or ground‐glass nuclei	Excellent prognosis with the 20‐year OS greater than 90% [156] Metastases most commonly involve cervical lymph nodes and, less commonly, the lungs [157]
OCA	Minimally invasive Encapsulated angio‐invasive Widely invasive	Mitochondrial DNA mutations [158, 159] widespread chromosome losses [106, 107] Other mutations: *RAS*, *EIF1AX*, *TERT*, *TP53*, *NF1*, *CDKN1A* [30]	≥75% oncocytes Large size, deeply eosinophilic and granular cytoplasm, and large nuclei with prominent nucleoli Nuclear features of PTC and high‐grade features are absent	Common radioactive iodine‐refractory [160, 161, 162] Distant metastasis: 15–27%; Extensive vascular invasion: 40% [163] 5‐year OS: 91% (M0) vs 24% (M1) [164]
Follicular‐derived carcinomas, high‐grade	PDTC DHGTC	*RAS* (PDTC)/BRAF (DHGTC) mutations [165, 166] *TERT*/*TP53* (late events) [166, 167] *DICER1* (adolescent PDTC) [168]	PDTC: Solid/trabecular/insular growth; Partially lost cytological and architectural differentiation, without PTC nuclear; At least one of the following characteristics: convoluted nuclei, ≥3 mitosis per 10 high‐power fields (HPF), and tumor necrosis DHGTC: Any DTCs with ≥5 mitoses per 2 mm^2^ and/or tumor necrosis	5‐year OS: 75%; 10‐year OS: 54% 20‐year OS: 28% [169]
ATC	ATC SCC	*BRAF* and *RAS* mutation TP53/CDKN2A MMR‐deficient [170, 171, 172]	Loss of differentiation; Presenting epithelioid and spindle cell areas	Median OS: 6.5 months [173] 55% displaying local invasion and/or distant metastasis at diagnosis [174]

*Abbreviations*: FTC, follicular thyroid carcinoma; IEFVPTC, invasive encapsulated follicular variant papillary carcinoma; PTC, papillary thyroid carcinoma; OCA, oncocytic carcinoma of thyroid; OS, overall survival; DHGTC, differentiated high‐grade thyroid carcinoma; PDTC, poorly differentiated thyroid carcinoma; ATC, anaplastic thyroid carcinoma; DTC, differentiated thyroid carcinoma; SCC, squamous cell carcinoma.

##### 4.1.3.2 Papillary Thyroid Carcinoma

**FIGURE 5:**
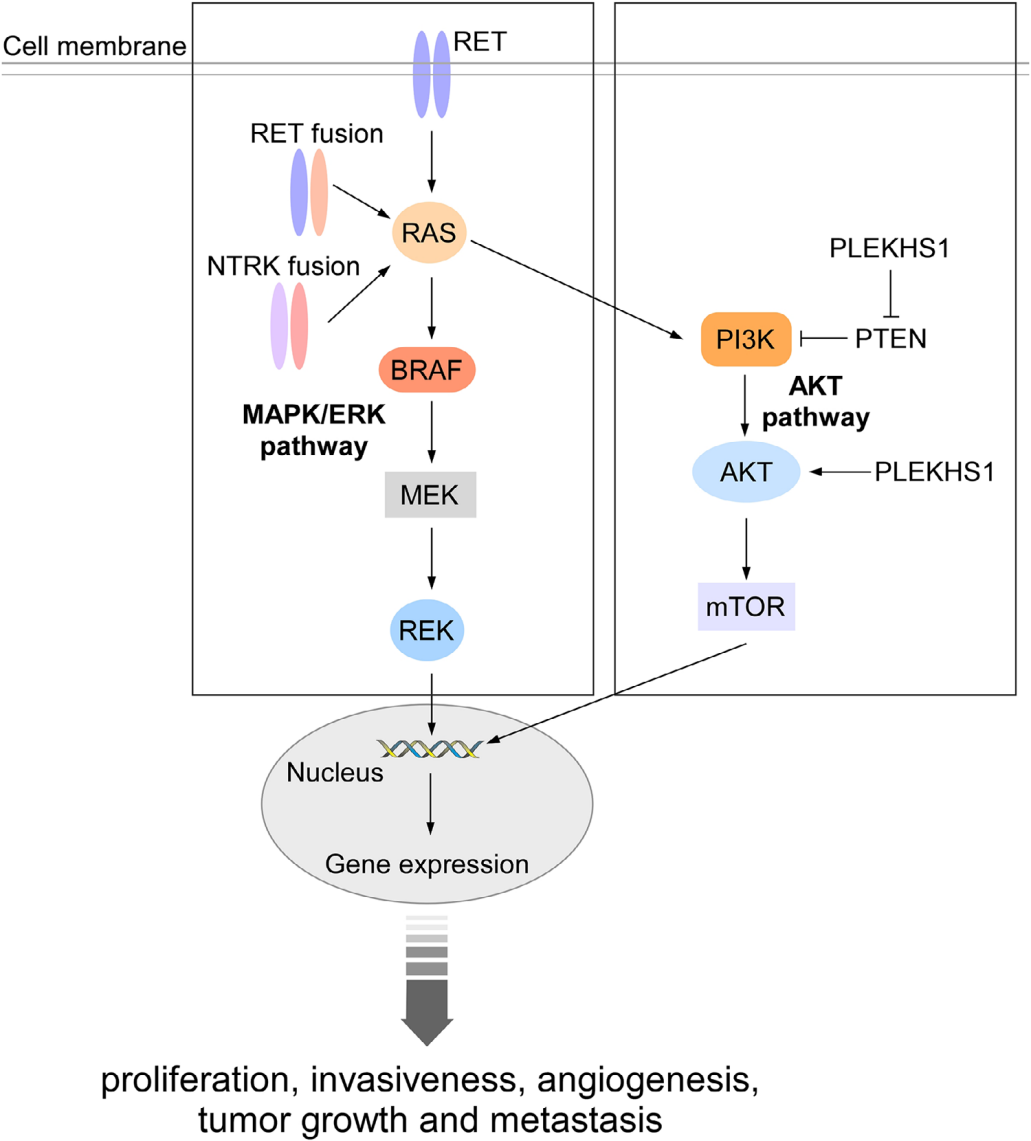
MAPK and AKT signaling pathway in follicular‐cell derived thyroid carcinoma. Upon activation by various fusion genes or mutations, receptor tyrosine kinases (RTKs) such as RET and TRK1/3 form dimers and initiate downstream signaling. This involves RAS‐mediated stimulation of the MAPK pathway, as well as PIK3‐dependent activation of the PI3K/AKT cascade. PLEKHS1 also leads to AKT pathway activation. *PTEN* negatively regulates PI3K‐mediated activation of the oncogenic PI3K/AKT pathway. Both pathways collectively promote cell proliferation, angiogenesis, and migration.

PTC, constituting 65–93% of global thyroid malignancies [30], represents the most common follicular cell‐derived cancer among adult and pediatric populations. The rising incidence rates observed worldwide predominantly reflect increased detection of small, low‐risk PTCs that might never cause symptoms or require therapy [26, 175, 176]. The fifth edition WHO classification recognizes several histopathological subtypes: infiltrative follicular, tall cell, columnar cell, hobnail, solid, diffuse sclerosing, warthin‐like, and oncocytic (Table 1). *BRAF*‐driven PTCs show morphologically less follicular differentiation than *RAS*‐driven FTC [128]. PTC pathogenesis is predominantly driven by MAPK signaling pathway alterations, with activating point mutations and chromosomal rearrangements constituting the principal oncogenic drivers (Figure 5) [128, 177, 178, 179, 180]. Histopathologic analysis reveals that *BRAF V600E*, the predominant genetic lesion in classical PTC, displaying papillary growth patterns and follicular‐structured invasive tumors, accounts for 60% of documented pathogenetic mutations [128], and are specifically overrepresented in the tall cell, columnar cell and hobnail subtypes−the PTCs with higher risk of lymph node metastasis, loco‐regional recurrences, distant metastases, and poor outcomes [96]. This mutation causes activation of the MAPK signaling pathway, which in turn stimulates extracellular signal‐regulated kinases transcriptional programs, leading to increased proliferation, angiogenesis, and invasiveness, as well as downregulating transcription of genes responsible for thyroid differentiation. (Figure 5) [96]. Second in frequency are telomerase reverse transcriptase (*TERT*) promoter mutations, occupying approximately 10% of all mutations in PTCs, which serve as robust biomarkers for predicting tumor aggressiveness and adverse clinical outcomes and are associated with aggressive clinical progression [134, 142, 143, 144, 145, 146, 147, 148]. Intriguingly, 13% of cases maintain undefined mutational etiology despite comprehensive genomic characterization [128, 181, 182]. Clinical progression patterns demonstrate divergent metastatic potential: lymph node involvement occurs in 25–30% of patients, whereas distant metastases (primarily pulmonary and osseous) manifest in 5% of cases [157].

##### 4.1.3.3 Oncocytic Thyroid Carcinomas

Oncocytic thyroid carcinomas (OCA) is a malignant follicular‐derived tumor comprising ≥75% oncocytes and lacking PTC nuclear features. Histologically, it shows follicular‐patterned oxyphilic cells with abundant mitochondria and prominent nucleoli (Table 1) [183]. OCA constitutes 3–7% of follicular cell‐derived cancers [30, 184] and includes several clinicopathological variants [185, 186]. OCAs are classified as minimally invasive, encapsulated angio‐invasive, or widely invasive. Evaluation requires assessment of mitotic activity and necrosis.

Clinically, OCAs are more aggressive than typical FTCs. They are often refractory to radioiodine and present with higher locoregional recurrence and distant metastases (15–27%, rising to >40% with vascular invasion) [160, 161, 162, 163]. The 5‐year overall survival varies by stage: 91% (M0) versus 24% (M1) [164].

Molecularly, OCAs exhibit mitochondrial DNA mutations disrupting complex I function [158, 159], leading to metabolic shifts toward glycolysis [187]. They often show chromosomal loss through near‐haploidization and whole‐genome duplication, with retention of heterozygosity on chromosome 7 [106, 107]. Somatic mutations affect MAPK (*NRAS*, *NF1*), cell cycle (*CDKN1A*, *TP53*), chromatin (*DAXX*, *EIF1AX*), and telomere maintenance (TERT promoter) pathways [30].

##### 4.1.3.4 High‐Grade Follicular Cell‐Derived Carcinomas

The fifth WHO classification introduces two distinct high‐grade follicular cell‐derived carcinomas: PDTC and differentiated high‐grade thyroid carcinoma (DHGTC). These tumors represent an intermediate stage between indolent DTC and aggressive ATC [96].

PDTC exhibits solid, trabecular, or insular architecture. Diagnosis requires either tumor necrosis or ≥3 mitoses per 10 high‐power fields (∼2 mm^2^) in necrosis‐free areas [188]. DHGTC, by contrast, retains papillary nuclear features or follicular architecture but meets high‐grade thresholds: ≥5 mitoses per 10 HPF and/or tumor necrosis [166, 167, 168].

Molecularly, PDTCs often carry *RAS* mutations, consistent with origins from FTC or encapsulated FVPTC. DHGTCs, typically evolving from PTC, harbor *BRAF V600E* mutations and PTC‐related gene fusions [165, 166]. Both accumulate late‐stage mutations such as *TP53* and *TERT* promoter, which are associated with aggressive behavior and distant spread [166, 167].

In adolescents, PDTC frequently involves DICER1 mutations, suggesting miRNA dysregulation as a unique driver in pediatric cases [168].

These tumor types are summarized in Table 1, which details histopathological features, molecular profiles, and clinical outcomes of major follicular‐derived thyroid carcinomas, aiding diagnostic precision and treatment planning.

##### 4.1.3.5 ATC and Squamous Cell Carcinoma

ATC, representing <1% of thyroid cancers, is reclassified in the fifth WHO edition alongside squamous cell carcinoma, a newly defined subtype sharing follicular cell origin, frequent *BRAF* mutations, and similarly poor outcomes [96, 170, 189]. These tumors are the most aggressive thyroid malignancies, with 45% of patients presenting with distant metastasis at diagnosis and a 5‐year cause‐specific survival rate of only 8% [190].

Molecular tracing confirms that ATC arises via dedifferentiation from pre‐existing DTCs (e.g., DHGTC, PDTC), retaining driver mutations from the original tumor. PTC/DHGTC‐derived ATCs commonly harbor *BRAF V600E* and *TERT* promoter comutations, while FTC/EFVPTC‐derived ATCs show *RAS* and *TERT* alterations. Progression is marked by additional inactivation of *TP53* and deletion of *CDKN2A/B*, disrupting cell cycle control [166, 170]. A subset of ATCs shows mismatch repair deficiency and high tumor mutational burden, usually involving *MLH1/MSH2* mutations. Although their clinical relevance remains unclear, they may define a distinct immunogenic subset [170, 171, 172]. Routine screening for *BRAF V600E* has become essential in ATC management. Clinical trials show that combined BRAF/MEK inhibition (e.g., dabrafenib + trametinib) leads to rapid tumor regression in *BRAF*‐mutant ATC, prompting guideline recommendations for immediate molecular or immunohistochemical testing [191].

### 4.2 Medullary Thyroid Carcinoma

MTC is a rare neuroendocrine tumor of parafollicular C‐cells, accounting for ∼1–2% of thyroid cancers [192]. It occurs in sporadic (75–80%) and hereditary (20–25%) forms. Sporadic MTC usually presents as a unifocal tumor in adults aged 40–60 years, with frequent early lymph node involvement (central: 11–86%; lateral: 11–93%) [193, 194, 195, 196]. Hereditary MTC is associated with bilateral, multifocal tumors and C‐cell hyperplasia, often as part of MEN2 syndrome, with autosomal dominant inheritance [197].

Clinically, MTC often presents with cervical lymphadenopathy. Around 70% of palpable tumors show nodal metastases at surgery. Systemic symptoms like flushing or diarrhea may suggest distant spread [195].

*RET* proto‐oncogene mutations drive hereditary MTC, with >100 variants showing genotype–phenotype correlations [198]. Somatic *RET* mutations are found in ∼50% of sporadic cases, while *HRAS*, *KRAS*, or rarely *NRAS* mutations occur in *RET*‐wild‐type tumors [199, 200, 201, 202].

The fifth WHO edition introduces a grading system: tumors are considered high‐grade if they exhibit tumor necrosis, ≥5 mitoses/2 mm^2^, or Ki67 index ≥5% [203]. Due to focal necrosis, adequate sampling is critical. This grading has prognostic significance regardless of germline *RET* status, though its predictive value for somatic *RET* mutations remains uncertain.

Future directions include clarifying the prognostic impact of grading in sporadic *RET*‐mutant MTC, identifying early metastasis biomarkers, and improving RET inhibitors for aggressive disease. Comprehensive genomic profiling may reveal new targets, especially in RET/RAS‐negative tumors. For hereditary MTC, optimizing surveillance and genotype‐based care will enhance personalized management [96, 204, 205].

### 4.3 Salivary Gland‐Type Neoplasms and Thymic Tumors Within the Thyroid

Salivary gland‐type and thymic tumors are exceptionally rare in the thyroid. Among salivary‐type neoplasms, the main subtypes include mucoepidermoid carcinoma (MEC), its mucinous variant, and secretory carcinoma (SC).

MEC consists of mucinous, intermediate, and squamoid cells in solid or cystic arrangements. It typically shows immunoreactivity for cytokeratin and p63, but is negative for Ctn and neuroendocrine markers. Follicular markers like Tg, TTF‐1, and PAX8 show variable expression. Though *MAML2* rearrangements have been detected, they are less frequent than in salivary MEC [206]. The histogenesis remains unclear, but associations with PTC, follicular carcinoma, oncocytic carcinoma, and lymphocytic thyroiditis support a hypothesis of squamous metaplasia as the precursor lesion [207].

SC, previously known as mammary analogue SC, is composed of eosinophilic tumor cells with vacuolated cytoplasm. It expresses GATA3, mammaglobin, and S100, but is negative for Tg, TTF‐1, and PAX8 [208, 209]. The pathognomonic *ETV6::NTRK3* fusion aids diagnosis and may predict response to TRK inhibitors [210, 211]. SC of the thyroid appears more aggressive than its salivary counterpart, with up to 30% recurrence or metastasis [207, 211, 212, 213].

Thymic tumors include intrathyroidal thymomas, thymic carcinomas, and spindle epithelial tumors with thymus‐like elements. Terminology has evolved; “carcinoma showing thymic‐like differentiation” is now classified as intrathyroid thymic carcinoma (ITC) under the WHO fifth edition guidelines [96]. While definitive molecular markers are lacking, TERT promoter mutations may distinguish ITC from mediastinal thymic counterparts [214].

These rare tumors illustrate evolving thyroid tumor taxonomy, highlighting the role of morphology, immune‐profiling, and molecular genetics in classification and therapeutic guidance.

### 4.4 Thyroid Tumors of Uncertain Histogenesis

The WHO fifth edition introduces “thyroid tumors of uncertain histogenesis,” including entities that lack definitive follicular, C‐cell, or thymic lineage based on molecular and immunophenotypic profiles. The cribriform‐morular thyroid carcinoma (CMTC), previously classified as a PTC variant, is now considered a separate tumor type due to its distinct molecular and immunophenotypic features [96]. CMTC is driven by Wnt/β‐catenin pathway alterations, particularly *APC* mutations, and lacks common mutations like *BRAF V600E*, *RAS*, or *PIK3CA* [215, 216, 217, 218, 219]. Immunohistochemically, CMTC lacks Tg and PAX8, with TTF‐1 positivity restricted to cribriform areas, but show CD5, CK5, and CDX2 positivity. These features support its reclassification as a tumor of uncertain histogenesis.

Sclerosing MEC with eosinophilia (SMECE) is another rare entity previously confused with conventional MEC. It is characterized by mucoepidermoid differentiation within a dense lymphoeosinophilic stroma [102, 212]. SMECE lacks expression of follicular markers (PAX8, Tg), and *MAML2* rearrangements are absent [220]. While TTF‐1 is variably expressed, its role is uncertain. SMECE commonly expresses p63, suggesting derivation from solid cell nests, although this remains speculative [221]. Both tumors emphasize how molecular profiling reshapes classification by identifying lesions that diverge from traditional thyroid lineages. As technologies like single‐cell sequencing advance, clearer lineage definitions and refined classification will likely emerge.

## 5 Long‐Term Management and Treatment

Treatment of thyroid cancer should be based on the final diagnosis and individualized risk assessment.

### 5.1 Active Surveillance

Despite diagnostic advances, overdiagnosis and overtreatment remain challenges in thyroid cancer management. Over 90% of thyroid nodules are euthyroid and benign at diagnosis, making AS a safe option for selected patients [222]. Current guidelines recommend AS for nodules ≤1 or <1.5 cm without concerning ultrasound features (e.g., extrathyroidal extension, nodal, or distant metastasis) [223].

The risk of malignant transformation in initially benign nodules diagnosed by FNA ranges from 2 to 7%, with some studies reporting as low as 1.1% [91, 224]. In a cohort of 1395 patients, 340 underwent AS while 1055 had surgery. Nodule growth ≥3 mm was seen in 6.4% at 5 years and 15.9% at 10 years; lymph node metastasis occurred in 1.4% (5 years) and 3.4% (10 years) [225]. It is worth emphasizing that none of the 109 AS patients who later had surgery experienced recurrence. Other independent studies support these findings, demonstrating that AS leads to comparable outcomes while avoiding unnecessary interventions [188, 226, 227]. Nevertheless, clinicians must monitor for progression. Growth or new suspicious features warrant re‐evaluation and possible treatment. AS provides a balanced approach, combining close observation with timely intervention—minimizing overtreatment while maintaining oncologic safety.

### 5.2 Surgery

Surgery remains the definitive treatment for enlarging nodules or those impairing the quality of life. It achieves durable remission in over 50% of low‐risk, well‐differentiated thyroid cancers [14, 62]. A comprehensive preoperative ultrasound is essential to assess thyroid morphology and cervical lymph nodes. For unilateral, WTD ≤4 cm, lobectomy is preferred, offering outcomes equivalent to total thyroidectomy with lower complication rates (e.g., permanent hypoparathyroidism). Total thyroidectomy is reserved for bilateral tumors or those ≥4 cm [62]. Central/lateral neck dissection is indicated for confirmed nodal metastasis. Prophylactic dissection is limited to MTC or advanced DTC with extrathyroidal extension. Lateral neck dissection is typically considered in high‐risk MTC cases only. Over the past decade, minimally invasive techniques have gained ground in selected cases. These include percutaneous ethanol injection, RFA, and HIFU [228]. A meta‐analysis (*n* = 2599) found that RFA and microwave ablation offered superior symptom relief, cosmetic outcomes, shorter hospital stays, and fewer complications compared with surgery [229].

International societies now recommend ablation techniques for patients with well‐demarcated benign nodules ≥4 cm, showing steady growth or causing cosmetic/compressive symptoms [230, 231, 232, 233]. However, conventional surgery remains preferred for large goiters, toxic multinodular goiter, or multifocal disease, where extensive intervention is required [234]. The expanding role of nonsurgical modalities, supported by growing evidence and international guidelines, reflects the shift toward individualized and less invasive management strategies.

### 5.3 Adjuvant Therapy for Thyroid Cancer

RAI is used after total thyroidectomy to ablate remnant tissue, treat microscopic disease, or manage persistent high‐risk cases [62]. Current guidelines recommend a risk‐adapted approach: RAI is not routinely advised for low‐risk patients, as multiple trials show no significant benefit in recurrence reduction [235, 236, 237, 238]. Alternatively, high‐risk patients benefit from improved survival and reduced recurrence with RAI [239]. Use in intermediate‐risk cases requires individualized assessment [240].

Molecular features influence RAI responsiveness. Tumors with *RAS* mutations (e.g., FTC, FVPTC) often retain sodium‐iodide symporter function and RAI avidity. Conversely, *BRAF*‐mutant tumors typically show reduced RAI uptake due to symporter suppression, particularly in persistent disease [241]. TSH suppression therapy is also essential postoperatively. TSH promotes growth in both normal and DTC cells [242, 243]. Suppression reduces recurrence and disease‐specific mortality in high‐risk patients when TSH is maintained <0.1 mU/L [230]. Intermediate‐risk patients may benefit from moderate suppression (0.1–0.5 mU/L), although supporting evidence is less robust. Low‐risk patients do not require TSH suppression. Since C cells lack TSH receptors, this approach is ineffective for MTC [62, 244, 245].

Given risks such as atrial fibrillation and osteoporosis, TSH suppression must be tailored to risk‐benefit profiles. As molecular testing improves, new biomarkers may help guide RAI and TSH strategies for optimized patient outcomes.

### 5.4 Treatment of Advanced Thyroid Cancer

The treatment of radioiodine‐refractory (RAIR) DTC and recurrent or metastatic MTC requires careful assessment of disease kinetics, symptom burden, and potential organ involvement. While many thyroid cancers progress slowly, systemic therapy is considered when there is radiological evidence of progression within 14 months, symptomatic lesions, tumors ≥1.5 cm, or risk of functional impairment [246, 247, 248].

In selected cases, local treatments such as surgery or external beam radiotherapy may provide palliation and delay systemic intervention. From 2013 to 2015, several multikinase inhibitors (MKIs) were United States Food and Drug Administration (US FDA) approved: sorafenib and lenvatinib for RAIR DTC, and vandetanib and cabozantinib for MTC (Table 2). These agents primarily inhibit VEGFR‐mediated angiogenesis [249, 250, 251, 252]. Cabozantinib and lenvatinib have also shown benefit as second‐line therapies following sorafenib progression [253].

**TABLE 2:** FDA‐approved drugs for thyroid cancer.

Drugs	Targets	Indication	Response rate	References
Sorafenib	VEGFR, PDGFR, RET	Radioactive iodine refractory (RAIR) DTC	12.2%	[249]
Lenvatinib	VEGFR, PDGFR, FGFR, RET	RAIR DTC	64.8%	[250]
Vandetanib	VEGFR, PDGFR, RET	MTC	45%	[251]
Cabozantinib	VEGFR, RET, MET	MTC	28%	[252]
		Second‐line RAIR DTC	15%	[253]
Dabrafenib + trametinib	BRAF/MEK	BRAF‐mutated ATC	56%	[254]
Dabrafenib	BRAF	BRAF‐mutated RAIR DTC	35%	[255]
Larotrectinib	NTRK	NTRK‐fusion thyroid cancer	DTC: 86% ATC: 29%	[256]
Entrectinib	NTRK	NTRK‐fusion thyroid cancer	53.8%	[257]
Selpercatinib	RET	MTC with RET mutation	69.4%	[258]
		DTC/ATC with RET fusion	79%	[259]
Pralsetinib	RET	DTC/ATC with RET fusion	89%	[260]

Recent molecular advances have led to the development of selective targeted therapies. Selpercatinib and pralsetinib target *RET* alterations, while larotrectinib and entrectinib treat *NTRK* fusion‐positive tumors (Table 2). Dabrafenib, combined with trametinib, is approved for *BRAF V600E*‐mutated ATC and DTC (Table 2) [254, 255, 256, 257, 258, 259, 260]. These agents offer high response rates and are often better tolerated than traditional MKIs. Besides, for tumors without actionable mutations, immunotherapy is a promising option. In a phase II study, the PD‐1 inhibitor spartalizumab demonstrated a 19% response rate in ATC, with improved outcomes in PD‐L1‐positive patients (52.1% 1‐year survival) [261]. These findings suggest a potential role for immune checkpoint blockade in selected patients with aggressive disease.

As summarized in Table 2, US FDA‐approved systemic therapies now span multiple molecular targets, enabling personalized treatment for advanced thyroid cancer. Integration of genomic profiling into routine care continues to shape treatment decisions, particularly for aggressive forms like ATC. Ongoing research will further define optimal sequencing, combination strategies, and predictors of therapeutic response [261].

## 6 Clinical Staging and Risk Stratification

Thyroid cancer prognosis is primarily determined using the AJCC TNM staging system, which evaluates tumor size, patient age, regional lymph node involvement, local invasion, and distant metastases [4]. This system accurately predicts disease‐specific survival, but is less informative for assessing the risk of recurrence, especially in DTC. To address this, supplementary risk stratification models such as those from the ATA and ETA have been widely adopted. These classify patients into low‐, intermediate‐, and high‐risk groups, helping guide follow‐up intensity and decisions on adjuvant therapies like RAI and TSH suppression.

Recent studies suggest additional prognostic factors can refine risk assessment. One such factor is mitotic activity, which correlates with tumor aggressiveness in PTC. Although not included in current ATA guidelines, a single‐center analysis of 640 PTC cases (2018–2022) showed high mitotic index was associated with adverse histopathology and increased recurrence rates [262].

Furthermore, a large prospective Italian multicenter study (*n* = 4773) developed a data‐driven recurrence prediction model, incorporating clinical and demographic variables not captured by ATA risk categories. These include age, sex, BMI, family history, presurgical cytology, surgical approach, and whether the diagnosis was symptomatic or incidental [67]. The model demonstrated superior predictive accuracy compared with standard risk systems, highlighting the limitations of traditional models and the need for more individualized risk assessment tools.

As thyroid cancer care increasingly embraces precision medicine, future staging systems will likely integrate molecular markers (e.g., *BRAF*, *RAS*, *TERT* promoter mutations) with clinicopathological data. These markers, which also influence therapeutic decisions, may improve recurrence prediction and guide more tailored interventions.

In summary, evolving risk models reflect the biological heterogeneity of thyroid cancer and reinforce the importance of integrating both clinical and genomic data to enhance prognostic accuracy and patient‐specific care planning.

## 7 Conclusion and Prospects

Despite the extensive advances in thyroid cancer diagnostics and therapeutics over recent decades, several critical gaps remain in our understanding and management of the disease. One pressing concern is the persistent challenge of differentiating indolent from aggressive tumors at the time of diagnosis, particularly among tumors with follicular architecture or indeterminate cytology. While the adoption of molecular profiling, including detection of *BRAF V600E*, *TERT* promoter mutations, and *RAS* family mutations, has greatly improved diagnostic precision, the clinical implications of many rare mutations and gene fusions remain unclear. For instance, in follicular‐patterned lesions or encapsulated variants, accurate classification often depends on nuanced histologic interpretation and complete capsular assessment [96], which are not always feasible in preoperative settings. Future studies should aim to develop noninvasive molecular classifiers, potentially incorporating ctDNA or miRNA panels, that can reliably predict tumor behavior before surgery. And the reproducible molecular diagnosis criteria is need to be standardized to aid the early identificatioln of high‐risk disease.

Ultrasound remains the cornerstone of thyroid cancer diagnosis and risk stratification. Another critical area for development is the standardization and global harmonization of ultrasound‐based RSSs. Although TI‐RADS variants such as ACR‐TIRADS, EU‐TIRADS, and C‐TIRADS have demonstrated strong performance in specific populations, their interobserver variability, inconsistent terminology, and limited incorporation of clinical parameters continue to limit their widespread applicability. Emerging initiatives such as the I‐TIRAD aim to unify sonographic criteria and improve reproducibility across diverse clinical settings [68]. Additionally, the integration of machine learning algorithms and AI‐assisted imaging analytics may enhance the diagnostic value of ultrasound by reducing subjective variability and enabling personalized malignancy risk estimates. Early‐stage research utilizing deep convolutional neural networks for thyroid nodule classification has shown promising diagnostic accuracy [263] suggesting that AI‐assisted decision support tools may become a standard adjunct in thyroid nodule evaluation. The future of thyroid cancer imaging will likely involve the integration of harmonized sonographic descriptors, machine learning‐assisted pattern recognition, and predictive analytics to provide individualized malignancy risk estimates. Efforts to incorporate patient demographics, serum markers, and clinical parameters into imaging models are already underway and hold promise for enhancing diagnostic precision while reducing unnecessary interventions.

In parallel, a growing body of literature has begun to question the economic and psychological burdens of overtreatment. Several population‐based studies, including those from Korea and China, have identified a disconnect between rising thyroid cancer incidence and stable or declining mortality, highlighting the epidemic of overtreated low‐risk cancers [9, 10, 11, 15, 16, 264]. This phenomenon is not limited to affluent nations, as similar trends have been reported in middle‐income countries due to the increased accessibility of high‐resolution imaging [265]. AS has emerged as a rational alternative for select low‐risk patients, yet its adoption varies considerably across institutions and cultures [266, 267]. More data are needed on long‐term outcomes, patient‐reported quality of life, and cost‐effectiveness of surveillance versus intervention, especially in diverse healthcare systems.

Therapeutically, the era of precision oncology has opened novel opportunities, but challenges remain in defining optimal sequencing strategies, managing resistance, and mitigating toxicity. For instance, while *RET* inhibitors (e.g., selpercatinib, pralsetinib) and *NTRK* inhibitors (e.g., larotrectinib, entrectinib) show impressive efficacy in *RET*‐ or *NTRK*‐altered tumors, their benefit is limited to molecularly selected populations [76]. Moreover, secondary resistance mutations and dose‐limiting adverse effects may curtail their long‐term utility. Ongoing clinical trials are investigating combination regimens, including dual‐targeted therapy and immune checkpoint inhibitors, for advanced thyroid cancers. Particularly in ATC, early‐phase trials combining BRAF/MEK inhibitors with immunotherapy have demonstrated encouraging responses, yet durable remission remains elusive in most cases [76]. There is a pressing need for real‐world studies, including biomarker‐based predictors of treatment response and toxicity profiles in elderly or comorbid populations, aiming to optimize efficacy while minimizing cumulative toxicity.

Looking forward, the integration of molecular diagnostics, artificial intelligence, and real‐world clinical data is expected to transform thyroid cancer care. Key priorities include the development of reliable predictive biomarkers, real‐time risk modeling tools, and individualized treatment algorithms that account for both tumor biology and patient preferences. Artificial intelligence applications in imaging interpretation, molecular data analysis, and decision support systems are likely to enhance diagnostic consistency and therapeutic accuracy. For aggressive subtypes such as ATC, early identification through rapid genetic testing and prompt referral to specialized multidisciplinary teams remains critical to improving outcomes. Ultimately, the future of thyroid cancer management lies in precision medicine, which seeks to integrate genomics, clinical staging, imaging characteristics, and patient‐centered metrics to deliver targeted, timely, and evidence‐based care. Continued innovation, international collaboration, and equitable access to advanced diagnostic and therapeutic tools will be essential to ensuring improved outcomes across the spectrum of thyroid cancer.

## Author Contributions

Yu‐dong Li and Qing‐yao Ye performed the main text writing. Yan‐xing Chen contributed to the introduction writing. Xia‐rong Hu contributed the full text modification and supervision. All the authors have read and approved the final manuscript.

## Conflicts of Interest

The authors declare no conflicts of interest.

## Ethics Statement

This study was approved by the ethics committee of The Tenth Affiliated Hospital, Southern Medical University (Dongguan People's Hospital) (No. KYKT2022‐070), and conformed to the principles of the Declaration of Helsinki. All participating patients provided written informed consent.

## Supporting information



Table S1. The information of patients shown in Figure 2 and 3.

## Data Availability

The authors have nothing to report.
