# Supplementary material for: Thyroid Cancer: Pathogenesis, Clinicopathology, Diagnosis, and Management
Source: MedComm (2020). 2025 Oct 28;6(11):e70449. doi: 10.1002/mco2.70449 (PMC12559858; doi:10.1002/mco2.70449)
Supplement: Supplementary file 1 — Table S1. The information of patients shown in Figure 2 and 3. [file MCO2-6-e70449-s001.docx]

| **Patient** | **Age** | **Gender** | **TI-RADS** | **Histologic subtypes** | **Whether to obtain written informed consent form** | **Whether to obtain ethical approval** |
| --- | --- | --- | --- | --- | --- | --- |
| **Fig. 2A** | 34 | Female | 2 | Thyroid follicular nodular disease | Yes | Yes |
| **Fig. 2B** | 37 | Male | 5 | papillary thyroid carcinoma | Yes | Yes |
| **Fig. 2C** | 19 | Female | 4 | papillary thyroid carcinoma | Yes | Yes |
| **Fig. 2D** | 47 | Male | 1 | Thyroid follicular nodular disease | Yes | Yes |
| **Fig. 2E** | 58 | Male | 3 | Follicular adenoma | Yes | Yes |
| **Fig. 2F** | 26 | Female | 5 | papillary thyroid carcinoma | Yes | Yes |
| **Fig. 3A** | 37 | Female | 4b/ | papillary thyroid carcinoma | Yes | Yes |
| **Fig. 3B** | 45 | Female | 2 | Thyroid follicular nodular disease | Yes | Yes |
| **Fig. 3C** | 24 | Male | 3 | Follicular adenoma | Yes | Yes |

**Table S1. The information of patients shown in Figure 2 and 3.**

The patients were collected in The Tenth Affiliated Hospital, Southern Medical University (Dongguan people's hospital) from April 16, 2023, to September 19, 2023. Key inclusion criteria for the patients in Figure 2 were patients with thyroid nodule diagnosed by ultrasound. Key inclusion criteria for patients in Figure 3 were needed to determine the function status of thyroid nodules for DTC patients. The patients with insufficient clinical data were excluded.
